# Profiling microbial communities in an extremely acidic environment influenced by a cold natural carbon dioxide spring: A study of the Mefite in Ansanto Valley, Southern Italy

**DOI:** 10.1111/1758-2229.13241

**Published:** 2024-02-26

**Authors:** Olga De Castro, Mariano Avino, Federica Carraturo, Emanuela Di Iorio, Donato Giovannelli, Michele Innangi, Bruno Menale, Nicolina Mormile, Jacopo Troisi, Marco Guida

**Affiliations:** ^1^ Department of Biology University of Naples Federico II Naples Italy; ^2^ Botanical Garden Naples Italy; ^3^ Department of Biochemistry and Functional Genomics Sherbrooke University Sherbrooke Quebec Canada; ^4^ National Research Council Institute of Marine Biological Resources and Biotechnologies—CNR‐IRBIM Ancona Italy; ^5^ Department of Marine and Coastal Science Rutgers University New Brunswick New Jersey USA; ^6^ Marine Chemistry & Geochemistry Department Woods Hole Oceanographic Institution Woods Hole Massachusetts USA; ^7^ Earth‐Life Science Institute Tokyo Institute of Technology Tokyo Japan; ^8^ EnvixLab, Department of Biosciences and Territory University of Molise Contrada Fonte Lappone Pesche (IS) Italy; ^9^ European Biomedical Research Institute of Salerno (EBRIS) Salerno Italy; ^10^ Theoreo srl Montecorvino Pugliano (SA) Italy

## Abstract

The Ansanto Valley's Mefite, one of the Earth's largest non‐volcanic CO_2_ gas emissions, is distinguished by its cold natural carbon dioxide springs. These emissions originate from the intricate tectonics and geodynamics of the southern Apennines in Italy. Known for over two millennia for its lethal concentration of CO_2_ and other harmful gases, the Mefite has a reputation for being toxic and dangerous. Despite its historical significance and unique geological features, there is a lack of information on the microbial diversity associated with the Mefite's gas emissions. This study presents an integrated exploration of the microbial diversity in the mud soil, using high‐throughput sequencing of 16S rRNA (Prokaryotes) and ITS2 (Fungi), alongside a geochemical site characterisation. Our findings reveal that the Mefite's unique environment imposes a significant bottleneck on microbial diversity, favouring a select few microbial groups such as Actinobacteria and Firmicutes for Prokaryotes, and Basidiomycota for Fungi.

## Graphical abstract

Microbiological and geochemical characterisation of extremely acidic soils arising from the cold, natural carbon dioxide springs of the Mefite in the Ansanto valley (southern Italy).

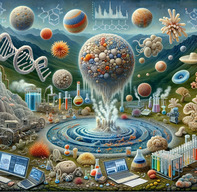

## INTRODUCTION

Extreme environments can be thought of as true laboratories of evolution (Di Iorio et al., 2023; Hallberg & Johnson, 2001; Johnson, 1998; Tobler et al., 2018; Wall & Virginia, 1999). The effects of extreme temperature, pH, salinity, and/or pressure, push species to their limits of survival. This selection process favours highly specialised communities with genetic and physiological adaptations to these extreme conditions (Di Iorio et al., 2023; Hallsworth, 2019; Takai, 2019). The vast majority of known extremophiles are prokaryotes (Archaea and Bacteria). These organisms have garnered significant research interest due to their production of enzymes active in extreme environments, which may have wide‐ranging industrial, medical, and economic benefits for humans (Atanasova et al., 2021; Shrestha et al., 2018; Siddiqui, 2015). Eukaryotic extremophiles have also been recognised, particularly plants that exhibit tolerance to drought, salinity, and extreme temperatures. Studying their physiology and response to such conditions is pertinent for agricultural advancements, especially in the context of climate change and soil salinisation (Carfagna et al., 2021; De Castro et al., 2018; Wang et al., 2003). Moreover, for example, plants have been studied for their ability to survive extreme cold of high mountain ecosystems (Larcher et al., 2010) as well as in enviornments like coastal sandy habitats that present challanging abiotic contidions (e.g., high temperature, salinity, shifting sand, strong winds, and radiation (De Castro et al., 2016, 2021). Animals, too, are subjects of interest for their adaptations to extreme environments, such as inhabiting very cold climates (Gertrudes et al., 2017), with these adaptations having significant relevance in the field of veterinary medicine (Irwin & Baird, 2004).

Across the globe, environments with extremely low pH values (i.e., < 3) are relatively common and often associated with volcanic activity and mining operations (Iovinella et al., 2020; Johnson, 1998; Merino et al., 2019). Acidic environments present numerous challenges to organisms, including the presence of noxious gases (e.g., H_2_S and CO_2_) and high concentrations of toxic elements (e.g., Al, Hg or As) (Meier et al., 2017; Quatrini & Johnson, 2018). Although some acidic environments can support vertebrates such as fishes, with certain species like those in the genus *Galaxias* tolerating pH levels just below 4 (Collier et al., 1990), most such habitats have a community composed of Rhodophyta, Archaea, Bacteria, and Fungi. The relative contribution of each to the total diversity is ultimately regulated by temperature. Extremophilic red algae, as *Galdieria* spp., have been extensively studied and found to be almost ubiquitous in extremely acidic environments worldwide, with a pH below 3 (Cozzolino et al., 2000; Eren et al., 2018; Schönknecht et al., 2013). Taxa like *Galdieria* spp. are exceptionally versatile, capable of utilising up to 50 different carbon sources, alternating between autotrophic and heterotrophic metabolism, and exploiting various nitrogen sources, depending on the pH of their environment (Iovinella et al., 2020). Archaea and Bacteria in acidic environments flourish at temperatures above 50°C and can be as high as 70°C, represented by highly specialised taxa such as *Thermoplasma*, *Ferroplasma*, and *Acidithiobacillus*. These organisms are adept at thriving in habitats with extreme conditions of both temperature and pH (Crognale et al., 2018). Similarly, filamentous fungi, including basidiomycetes and ascomycetes, have been discovered in waters with pH values as low as 2 and high arsenic concentrations (Aguilera, 2013; López‐Archilla et al., 2004).

**FIGURE 1:**
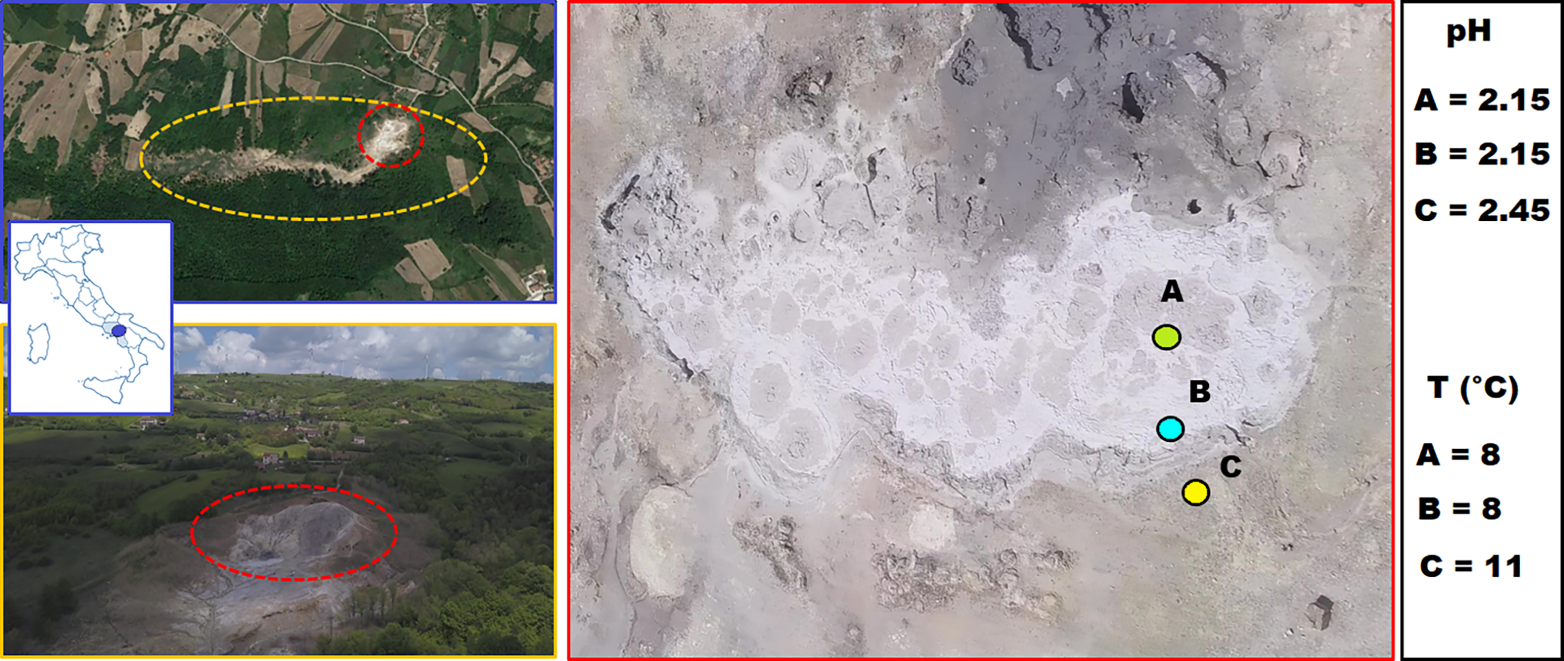
Map of the Mefite in Ansanto Valley (Avellino, southern Italy) and detail of cold lake seething with acid mud with the location of sampling sites (A, B, C) and relative information about temperature (*T*) and pH measured during samples collection. Sampling area with the three locations: (A) area of maximum emission of the bubbles (pH = 2.15 ± 0.006, *T* = 8°C); (B) near the shore (edge of the pond) (pH = 2.147 ± 0.006, *T* = 8°C); (C) about 50 cm from B (pH = 2.453 ± 0.006, *T* = 11°C).

Three major mechanisms contribute to the formation of hyperacid environments: (1) the direct degassing of strong acids associated with magmatic activity in proximity of open conduit active volcanoes, often leading to the creation of hyperacidic volcanic lakes (Mapelli et al., 2015); (2) the biological oxidation of sulphide and other reduced sulphur species, which produces sulphuric acid as a byproduct (Colman et al., 2018), a key process in the development of hyperacid environments associated with geothermal systems, mine drainage, and the exposure of sulphide minerals to oxygen; and (3) the presence of high concentrations of CO_2_ gases stemming from volcanic activity or other tectonic processes (Burton et al., 2013; Tamburello et al., 2018). The Mefite, located in the Ansanto Valley (southern Italy) is an example of the latter process given its extremely high concentrations of carbon dioxide along with the presence of non‐atmospheric nitrogen and hydrogen sulphide that are a consequence of tectonic processes (Chiodini et al., 2010), and it represents an exceptional site combining geology, archaeology, and biodiversity (Figure 1) (Sisto et al., 2020). Remarkably, Mefite represents one of the largest non‐volcanic gas emission areas, specifically a cold natural carbon dioxide spring, as depicted in Supplementary Video S1 by Di Iorio et al. (2023), associated with sedimentary and tectonic phenomena (Chiodini et al., 2010; Pischiutta et al., 2013; Sisto et al., 2020). In addition, Mefite is a mofette with a long and storied history of activity, as noted by historical figures such as Cicero in 44 B.C.E. or Vergil between 29 and 19 B.C.E. The gas emissions have been continuously documented over the past centuries, with records dating back to Di Capua in 1683 and extending to more recent studies by Chiodini (2014) and Di Iorio et al. (2023). Such extraordinary gas emissions have been estimated to be 2000 Mg per day, primarily consisting of CO_2_ (98%), non‐atmospheric N_2_ (1.3%), H_2_S (0.33%), and CH_4_ (0.23%) (Chiodini et al., 2010). The life‐threatening concentration of CO_2_, coupled with harmful levels of H_2_S, renders the innermost part of the Mefite almost devoid of life and potentially deadly. This has led to a millennia‐old cult in the valley, as documented by Santoli (1783), Chiodini (2014), and Sisto et al. (2020). Nevertheless, preliminary evidence of microbial extremophile organisms has been observed in the mud pools within the valley, as reported in the literature (Albertano et al., 1991; Albertano et al., 1994; Pinto et al., 1992; Totàro‐Aloj, 1973, 1974). However, the biodiversity and geochemical characteristics at the core of the Mefite site have remained underexplored. This study unveils the microbiological and geochemistry of a globally unique and relatively unknown location. By bridging a significant knowledge gap, it offers fresh insight into the microbial diversity and geochemical dynamics within the Mefite, an unparalleled ecosystem. We have employed tag‐amplicon sequencing of Prokaryotes and Fungi along with geochemical analyses of the mud pool to provide an integrated overview of the microbial community in this extraordinary environment for the first time.

## EXPERIMENTAL PROCEDURES

### Study site

The Mefite in the Ansanto Valley, located in Rocca San Felice within the municipality of Avellino (40.974369° N, 15.145646° E, 620 m a.s.l.), presents a desolate and spectral landscape. Its lunar‐like appearance, characterised by clayey‐calcareous terrain, is depicted in Figure 1. The Mefite's origin is tied to the uplift of the Apennine chain during the Pleistocene, as described by Cinque et al. (1993) and Improta et al. (2014). It features a significant area of non‐volcanic gas emission where gases are continuously released at ambient temperature (Figure 1 and Supplementary Video S1 by Di Iorio et al., 2023) (Chiodini et al., 2010; Pischiutta et al., 2013; Sisto et al., 2020), entering also in contact with deep‐water from the Picentini Mountains (Ortolani et al., 1981). In the area of maximum emission at the Mefite site, a pool of bubbling mud releases a substantial flow of gas. This gaseous river courses downstream, paralleling the Bagni stream (cf., Di Iorio et al., 2023). The gases are emitted from an area of approximately 4000 m^2^ (Chiodini et al., 2010), predominantly from a core area in the southwest part of the valley, marked by several mud ponds. Gas concentrations in the air vary significantly due to the markedly uneven terrain of the Mefite. The copious gas emissions generate a relentless flow that descends through the Bagni valley, which can be lethal to humans and animals as chronicled historically (Brocchi, 1820; Capano et al., 2015; Chiodini, 2014; Di Capua, 1683; Gambino, 1991; Macchia, 1838; Maiuri, 1953; Santoli, 1783), and evidenced by animal carcasses observed during various field inspections for the authors.

The vegetation around the Mefite site is profoundly affected by the gas emissions, with only a limited number of vascular plants able to endure the extreme soil acidity (pH <3; Battaglini & Totàro‐Aloj, 1973; Totàro‐Aloj, 1973) and high concentration of CO_2_ and H_2_S in the air (Chiodini et al., 2010; Sisto et al., 2020). Among the species that thrive in the vicinity of the toxic gas flow, two Poaceae [*Phragmites australis* (Cav.) Trin. ex Steud. and *Agrostis canina* L.] is particularly prevalent (Haworth et al., 2010). Additionally, a noteworthy Leguminosae, an ecotype of *Genista tinctoria* L. historically referred to as *G. anxantica* Ten., has been observed (Di Iorio et al., 2023). Despite the harsh conditions in the Mefite, the specific physiological and morphological adaptations that allow these plants to survive have not been extensively studied. A preliminary investigation by Haworth et al. (2010) examined the impact of the Mefite site's gaseous emissions (CO_2_ and SO_2_) on the development of stomata in *A. canina*, finding no correlation between a reduced stomatal index and plant resistance, nor any inhibition of stomatal initiation due to SO_2_ fumigation. In the case of *Genista*, recent molecular ecology analysis by Di Iorio et al. (2023) has revealed a significant genetic distinction between the Mefite population of *G*. *tinctoria* and nearby populations, suggesting it may indeed be an ecotype.

Other research on Mefite's biodiversity indicates that several contributions have focused on the pedofauna in the past. Studies on soil fauna have revealed the presence of a diverse range of invertebrates, including arthropods (e.g., springtails, oribatid mites, hard ticks), insects (e.g., Coleoptera, Diptera, Hemiptera, Hymenoptera, Odonata, Psocoptera), nematodes, and tardigrades, both upstream and along the sides of the mephitic source. In contrast, a decline in biodiversity is observed downstream, likely due to a higher concentration of toxic fumes that flow along the Bagni stream (Battaglini & Arcamone, 1981; Battaglini & Carbone, 1981; Battaglini & Totàro‐Aloj, 1973). For a comprehensive understanding of the Mefite system, refer to Di Iorio et al. (2023).

#### Sampling methods and field measurements

In March 2019, mud samples were collected using sterile 25 mL plastic containers. The sampling took place around noon on a sunny day (T_min_ 7°C, T_max_ 13°C, humidity 37%, and wind speed 30 km/h), with favourable wind conditions ensuring that gas fumes do not stagnate in the sampling area. Samples were gathered from the centre of the main pond (A), its boundary (B), and the immediate vicinity (C), as illustrated in Figure 1. The temperatures of the samples were approximately 8°C for A and B, and 11°C for C (Figure 1). All samples were immediately sealed and kept under ice during transport to the laboratory. Subsequently, they were freeze‐dried and stored at −80°C for chemical and molecular biology analysis.

### Chemical analyses

#### pH determination

The pH of the mud samples was measured potentiometrically using a PC 700 pH metre (Eutech Instrument, Singapore) on non‐pretreated samples. For each sample, 2 g of mud were vortexed with 5 mL of 1 M KCl solution at 92 rcf for 2 h. Prior to pH measurement, the resulting mixtures were centrifuged at 2400 rcf for 5 min.

#### Trace element determination

Microwave‐assisted acid oxidative digestion with nitric acid (HNO_3_ ≥ 69% v/v TraceSELECT, Sigma‐Aldrich Milan, Italy) was performed at high temperature (up to 180°C) and pressure. Following digestion, trace element contents were analysed by inductively coupled plasma‐mass spectrometry (ICP‐MS) (Aurora M90, Bruker, MA, USA) against a multi‐element standard solution for ICP TraceCERT®, in 5% nitric acid (Sigma‐Aldrich Milan, Italy) and ultrapure deionised water with conductivity <0.06 μS/cm. The purity of argon used for plasma gas is 99.999%. The speed of the three‐channel peristaltic pump operated at 50 rpm for 40 s in pre‐flush condition and at 4 rpm during analysis. The plasma power level was 1400 W.

The quantified elements included Al, As, B, Ba, Cd, Co, Cr, Cu, Fe, Hg, Li, Mn, Mo, Ni, Pb, Sb, Se, Sr, V, and Zn. Prior to analysis, a 30‐min equilibration period was conducted to stabilise the plasma temperature and ion optics. Instrumental stability was verified by analysing a tuning solution containing all analytes of interest at least four times; analyses proceeded only if the relative standard deviation was <5% for each analyte. Metal concentrations in the unknown solutions, appropriately diluted within the calibration curve's concentration range, were determined using an external calibration curve prepared with five concentrations for each analysed element. Calibration standards were prepared in a 1% HNO_3_ matrix from their respective stock solutions. ICP‐MS analyses of each unknown were conducted in triplicate. Elements with a concentration below the instrument's detection limits were excluded from further analysis.

#### Anion determination

Anions (F^−^, Cl^−^, Br^−^, NO_2_^−^, NO_3_^−^, PO_4_^3−^, SO_4_^2−^) were measured after H_2_O extraction by adding 100 mL of MilliQ water to 5 g of sample and mechanically stirred overnight for 8 h. The final solution, filtered through 0.45 μm cellulose membrane was injected in the Ion Chromatography (IC) system (Shimadzu, Kyoto, JP) consisting of two pumps LC20AD XR, a degasser DGU20A3, an autosampler SIL20AD XR, an oven CTO10AS VP and a suppressed conductivity Detector CDD‐10AVP. Ion separation was achieved using an Allsep Anion 7 u (Grace, Aiken, SC, USA) using 0.85 mM NaHCO_3_ + 0.9 Na_2_CO_3_ water solution as a mobile phase. The chromatograms obtained were compared with those obtained from acid solutions of known concentration using calibration with the external standard method. For calibration, an external standard (Ultra Scientific Italia Srl, Bologna, Italy) was employed. The calibration procedure utilised linear regression techniques, which were based on chromatographic peak areas, to establish precise quantification for each of the target anions.

#### Total volatile content

Volatile content (including water) was determined by gravimetry. Two grams of untreated sample were dried at 110°C for 8 h, weighted using an EX124 Explorer balance (Ohaus, Parsippany, New Jersey, USA). Volatile substances were expressed as the percentage of weight loss.

#### Dissolved organic compounds (DOC)

DOC was measured on the same sample solutions used for IC analysis. DOC was determined using the Shimadzu TOC‐V system (Shimadzu, Kyoto, JP), against a standard solution of KHP at increasing concentrations. All analyses were conducted in triplicate.

#### Cellulose

Cellulose was determined according to a colorimetric method (Danise et al., 2018). Briefly, samples were pre‐treated with a solution of 8:2:1 (v/v/v) glacial acetic acid, distilled water, and nitric acid (69%) to dissolve most cell‐wall components, including hemicellulose and lignin. After several washings with distilled water and drying, samples were treated with 72% sulphuric acid. After partial dehydration of cellulose, anthrone (2 g/L dissolved in concentrated sulphuric acid) reacts with the remaining 5‐hydroxymethylfurfural forming a blue‐green complex peaking at 690 nm. The calibration was done using a stock solution of cellulose (0.1 g L^−1^ in 72% sulphuric acid). All analyses were conducted in triplicate.

### Molecular analyses

#### Microbial DNA extraction, PCR amplification, and high‐throughput sequencing

Strict procedures to avoid contamination of reagents and between samples were followed. DNA extractions and PCR setup were performed in two different cabinets: one HEPA‐filtered and UV‐irradiated for DNA extraction (vertical 700 laminar flow, ASALAIR) and another one UV‐irradiated PCR cabinets (aura PCR, BIOAIR) within a laboratory with dedicated different separate zones for the several procedures (DNA, pre‐ and post‐PCR laboratory). Different sets of pipettes with filter tips were employed for DNA extraction and PCR procedures.

Microbial community DNA was extracted in triplicate from 0.24 g (dry weight) of acid mud using a specialised protocol (Supplementary Appendix S1). This method, tailored for the matrix's unique characteristics, drew upon insights from prior studies (De Castro et al., 2015; Lever et al., 2015; Plassart et al., 2012). Several kits and alcohol precipitation methods were initially unsuccessful due to the mud's high secondary polysaccharide content, which interfered with DNA extraction by saturating columns and complicating resuspension in alcohol. The extracted DNA was then quantified with a Qubit 3 Fluorometer (Invitrogen, Thermo Fisher Scientific).

To assess the composition and diversity of Prokaryotes and Fungi in each sample, amplicon surveys were conducted on a portion of the 16S rRNA gene (V3–V4 regions) and the intergenic transcribed spacer (ITS2). The 16S rRNA gene was amplified using the barcoded primer sets Pro341F (5′‐CCT ACG GGN BGC ASC AG‐3′) and Pro805R (5′‐GAC TAC NVG GGT ATC TAA TCC‐3′) (Takahashi et al., 2014); for the ITS2 nuclear marker, ITS3 (5′‐GCA TCG ATG AAG AAC GCA GC‐3′) and ITS4 (5′‐TCC TCC GCT TAT TGA TAT GC‐3′) primers (White et al., 1990). Both the forward and reverse primers of 16S and ITS2 markers included Illumina overhang adapter sequences (forward: 5′‐TCG TCG GCA GCG TCA GAT GTG TAT AAG AGA CAG‐3′; reverse: 5′‐GTC TCG TGG GCT CGG AGA TGT GTA TAA GAG ACA G‐3′).

PCR was conducted using a 40 μL reaction mixture, which consists of approx. 1 ng of DNA template and Kodaq 2X PCR MasterMix (Applied Biological Materials Inc., ABM), along with 0.25 μM of each primer. The reaction conditions were as follows: an initial denaturation at 94°C for 3 min, followed by 35 cycles of 94°C for 30 s (denaturation), 55°C for 30 s (annealing), and 72°C for 30 s (extension). A final extension step was carried out at 72°C for 5 min.

A DNA‐free blank was used for each primer set, using the same procedure for the samples analysed. To ensure reproducibility, a duplicate was carried out for each amplicon set. Prior to the PCR reaction, to remove potential microbial DNA contaminants in the MasterMix and water, as suggested by Carroll et al. (1999), a pre‐treatment was carried out using 2.5 U of Bsp143I (Sau3AI) (Thermo Fisher Scientific). This involved incubating at 37°C for 30 min, followed by a deactivation step at 65°C for 20 min. Additionally, a positive control for the digestion reaction was carried out using 400 ng of pGem‐3Zf + plasmid DNA (Applied Biosystem, Thermo Fisher Scientific). Despite the negative blank showing no visible amplicons in electrophoresis and Qubit analyses, a nested PCR targeting only the Illumina overhang adapter sequences was conducted on 1 μL of the blank to check for possible micro‐contamination during the first PCR.

The amplicons were qualitatively assessed using  the UVIdoc HD5 gel documentation system (UVITEC) and quantitavely measured with a Qubit 3 Fluorometer. Subsequently, 25 μL of each amplicon (concentration >5 ng/μL) was sent to BMR Genomics s.r.l. (Padua, Italy) for sequencing on a MiSeq platform (2 × 300 paired‐end sequencing; 2 × 50,000 reads/sample) (Illumina).

#### Data analysis

MiSeq pair‐ends sequencing data from 16S rRNA and ITS2 regions were obtained from six samples (*n* = 6), each representing two replicates from sites A, B, and C. Two negative blanks were included in the analysis, one each for ITS2 and 16S rRNA data. The sequences underwent initial quality inspection, editing, and adapter removal using Trim Galore 0.6.1 (http://www.bioinformatics.babraham.ac.uk/projects/trim_galore/). Trim Galore functions as a wrapper script that automates the use of several software tools. Initially, it employs FastQC v0.11.9 to perform a quality check, summarising and visualising quality reports from raw sequence data (fastq files). This step enables users to identify and address sequence issues, such as trimming low‐quality ends or removing adapters. These corrections are executed in the subsequent step using Cutadapt v.2.8. Afterward, FastQC regenerates quality reports to verify the effectiveness of these interventions. In our study, Trim Galore was utilised with default settings, including a Phred quality score threshold of 20 and automatic adapter trimming, detecting Illumina universal adapter primers. Pair‐end reads were merged using PEAR v0.9.8 (Zhang et al., 2013). For the ITS2 region, ITSx v1.0.11 was used (Bengtsson‐Palme et al., 2013) to specifically isolate ITS2 sequences, excluding regions like 5.8S and LSU. Chimeras were identified and removed using VSEARCH v1.11.1 (Rognes et al., 2016), which involved running our sequences against a dedicated chimeric database using the UCHIME algorithm (Edgar et al., 2011). All subsequent analyses were conducted using QIIME v1.9.1 (Caporaso et al., 2010).

Sample normalisation was performed rarefying to a constant sum, corresponding in our case to the lowest read depth sample (the lowest common denominator), throwing away extremely low depth samples, which were not present in our study.

Operational Taxonomic Units (OTUs) were picked using the script pick_open_reference_otus.py from QIIME with the ‘–suppress‐align‐and‐tree’ flag employing the reference and taxonomy databases version ‘sh_refs_qiime_ver8_dynamic_02.02.2019’ (https://unite.ut.ee/repository.php#uchime) for ITS2, ‘Silva 138 SSU Ref NR 99’ for 16S rRNA (https://www.arb-silva.de/documentation/release-138/).

Silva database was reformatted for being used in QIIME1 using the software make_Silva_db (https://github.com/mikerobeson/make_SILVA_db).

Levels of contaminants were checked with SourceTracker v.0.9.5 (Knights et al., 2011), comparing the presence of OTUs in samples of interest and blanks. Shared picked putative contaminant OTUs between blank and samples were removed through QIIME1 following a procedure described in Sheik et al. (2018).

Alpha and beta‐diversity analyses were conducted using the QIIME script core_diversity_analyses.py. The resulting plots were generated for OTUs taxonomic assignments and their relative frequencies, broken by ranks, as well as group the significance for the categories tested. In the graphic plot, OTUs with a frequency of less than 1% were classified as *other*. Statistical analysis within QIIME was performed using the nonparametric test ANOSIM test with 9999 permutations (Fierer et al., 2010). This yielded the R statistic and an associated *p*‐value.

### Statistical analysis

Independent two‐tailed *t*‐tests were used to compare parameters between two groups, considering an *ɑ*‐value <0.05 as statistically significant. Concentration values below the limit of detection (LOD) were imputed as LOD/√2, following Hornung and Reed (1990). To explore covariance patterns between datasets, we employed the two‐block partial least squares (2B‐PLS) multivariate statistic. This approach is suitable for matrices with a low sample size and highly correlated variables (Barker & Rayens, 2003; Carrascal et al., 2009). Although originally designed for morphometric analyses, this technique has been used also in ecological research (Innangi et al., 2019, 2020; Parisi et al., 2021). In 2B‐PLS, covariance patterns between the two multivariate datasets can be represented by a scatterplot for the first axis of 2B‐PLS, where the *x*‐axis and the *y*‐axis represent the two matrixes, respectively. Correlation patterns, positive or inverse, within and between matrixes are identified through tables showing correlation values for both blocks. We conducted an analysis comparing Chemical Variables versus Prokaryotes OTUs and Chemical Variables versus Fungi OTUs. All analyses were done in R 4.1.0 (RDevelopment Core Team, 2021; https://www.R-project.org), using ‘plsdepot’ (Sanchez, 2012; https://cran.r-project.org/package=plsdepot) and ‘ggplot2’ (Wickham, 2016) packages. Results are reported as mean ± standard error.

## RESULTS

### Chemical analyses

#### Main chemical characteristics

All sites showed pH levels below 3, with marginally higher values only for site C (A: 2.15 ± 0.006, B: 2.147 ± 0.006, C: 2.453 ± 0.006) (Figure 1). Similarly, sites C showed higher values of volatile substances (%; A: 12.557 ± 0.732, B: 11.183 ± 0.598, C: 49.12 ± 0.563), DOC (mg/mL; A: 674 ± 4.359, B: 697.667 ± 10.44, C: 734.333 ± 6.692) and correlated cellulose (mg/g; A: 3.982 ± 0.127, B: 6.314 ± 0.445, C: 9.217 ± 0.613). The latter increased steadily from site A to site C.

#### Trace element analysis

**FIGURE 2:**
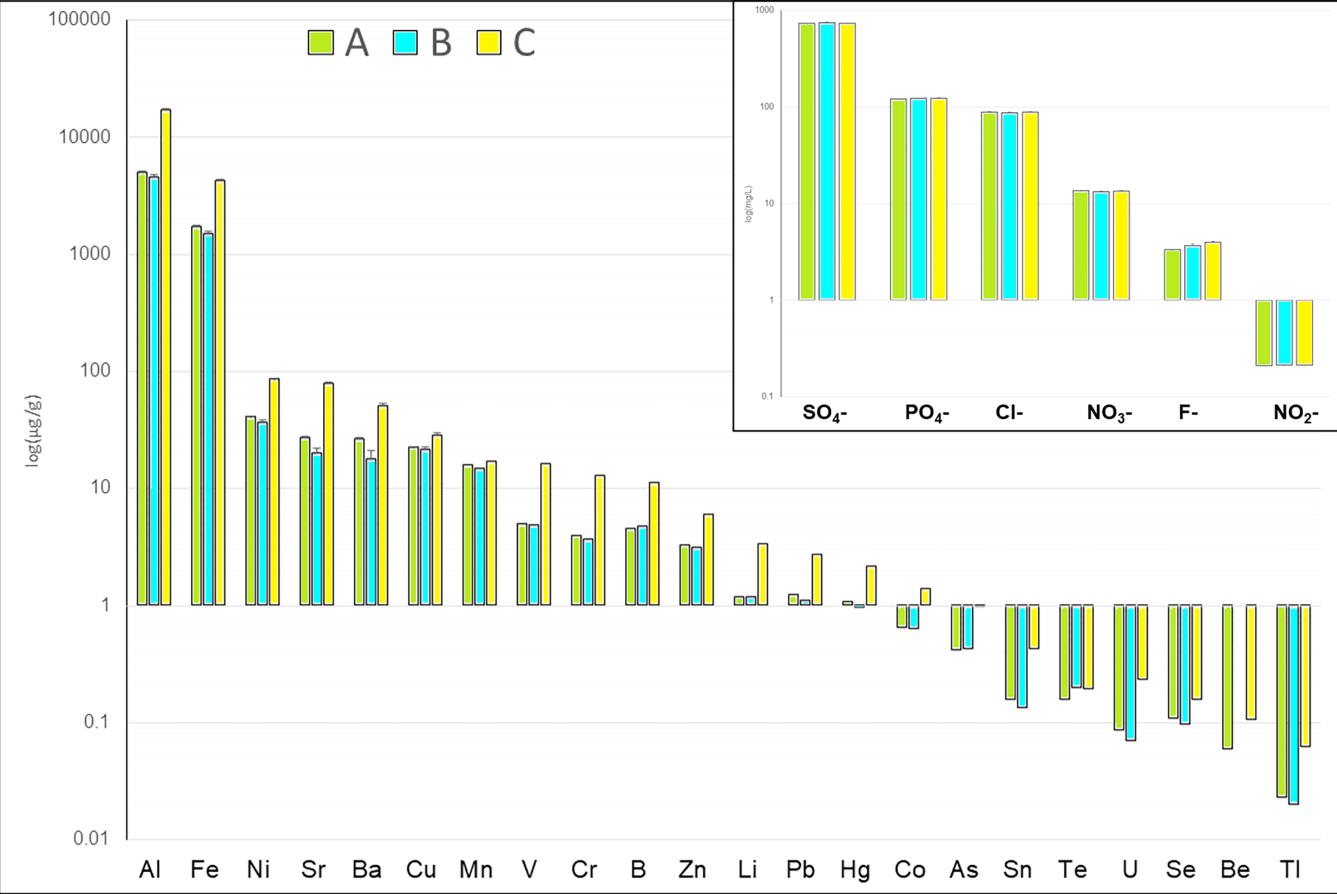
Trace element (μg/g) and anions (mg/L) concentration (above right) for the three sampling locations (sites A, B, C; Figure 1). Values are mean ± standard error of the mean (*N* = 3). Data are represented in logarithmic scale base 10.

The chemical characterisation of sites A, B, and C for each analysed sample is reported in Figure 2 (Supplementary Table S1 for the detailed values). Among all analysed trace elements, the majority of Mo, Ag, Cd, and Sb reads were below detection limits and were discarded from further analyses. Generally, across all sites, the elements with the highest concentration were Al, followed by Fe > Ni > Sr > Ba. Notably, Al's concentration was 3.6 times greater than that of Fe and between 160 and 280 times more than that of Ni, Sr, and Ba. Among the least concentrated trace elements, we detected Te > U > Se > Be > Tl, with Te being 5 times more concentrated than Tl. Among the three sites (A, B, and C; Figure 1), all elements showed the highest concentration at site C, except for Te, where the concentrations between sites B and C were similar. For Al and Fe, the concentration difference between sites A and C ranged from 2.5 and 3.5 times. Regarding the differences between sites A and B, almost all trace elements showed higher concentration at site A, with the notable exceptions of B, As, and Te, which were more concentrated at site B.

#### Anions

As depicted in Figure 2, the concentration of anions follows this order SO_4_^2−^ > PO_4_^3−^ > Cl^−^ > NO_3_^−^ > F^−^ > NO_2_. The differences in anion concentrations between sites were more subtle compared to those of trace elements, with all values being comparable (see Supplementary Table S2 for detailed values).

### Molecular analyses

Raw reads pairs of the fastq files contained approx. between 86,000 and 179,000 reads. The percentage of reads that included adapters varied from 15.4% and 69.4%, which were subsequently removed. The count of reads that did not pass a quality Phred score cutoff of 20 ranged between 2% and 22.4%. Reads pair per sample were assembled, and chimera sequences were detected and eliminated.

**FIGURE 3:**
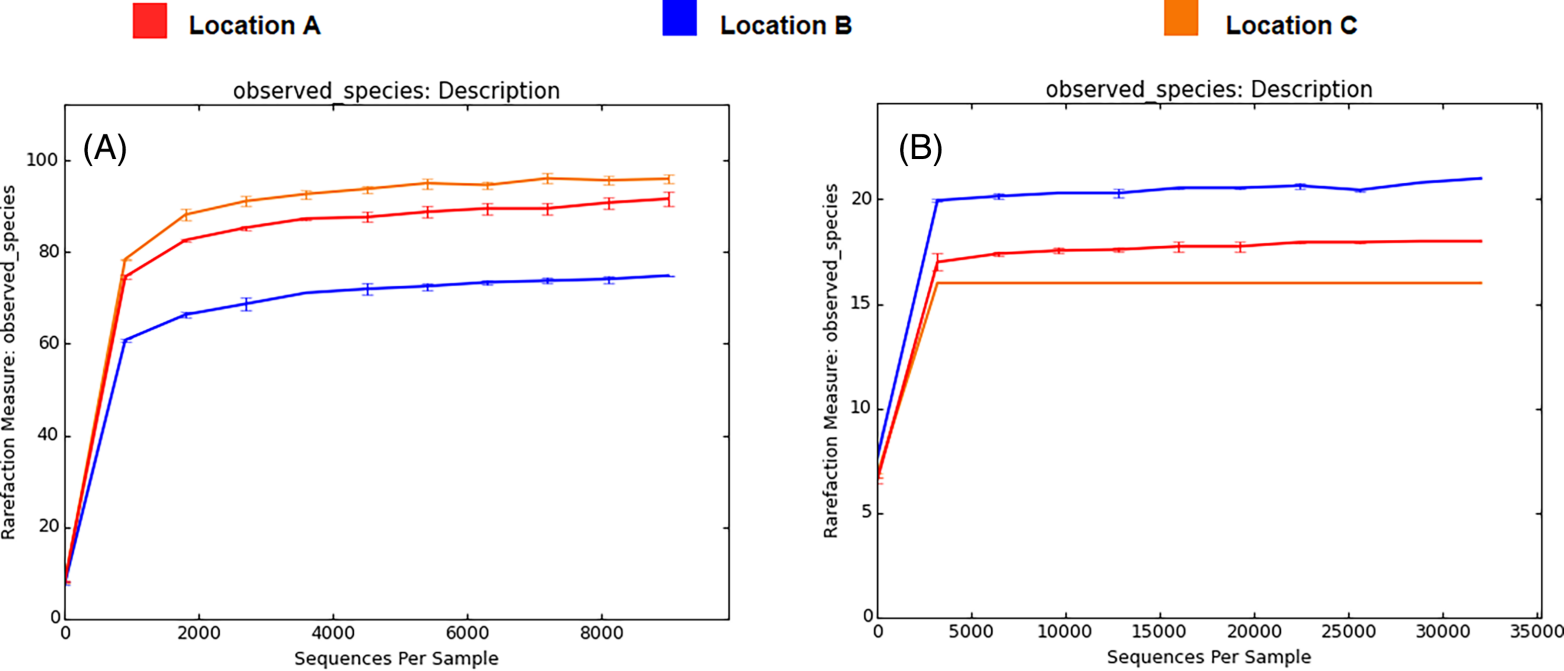
Alpha rarefaction plots per location (upper values): (A) Prokaryotes (16S rRNA marker); and (B) Fungi (ITS2 nrDNA marker).

**FIGURE 4:**
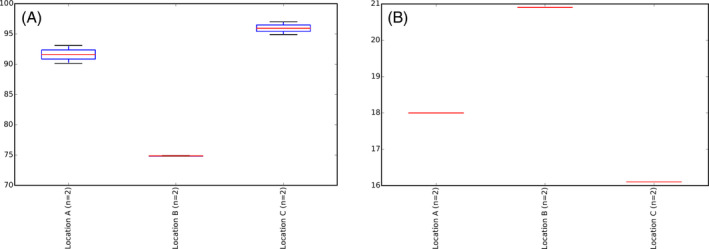
Alpha diversity box‐plots per location (upper values): (A) Prokaryotes (16S rRNA marker) (A); and (B) Fungi (ITS2 nrDNA marker).

The total read counts for 16S rRNA and ITS2 were 342,005 and 768,848, with a median value of 44,313 and 95,593, respectively. Following the removal of blank filtering (refer to methods), the OTUs count ranged from 9178 to 29,557 for 16S rRNA (with a rarefaction depth of 9000) and between 32,642 and 60,494 for ITS2 (with a rarefaction depth of 32,000). Rarefaction plots and corresponding box plots from the alpha diversity analysis are presented in Figures 3 and 4, employing ‘observed_otus’ as the statistical measure and examining the data by sample location. The alpha diversity analysis revealed how the number of OTUs per sample within the same location was comparable, yet it differed from samples in other locations, as shown in Figure 4.

**FIGURE 5:**
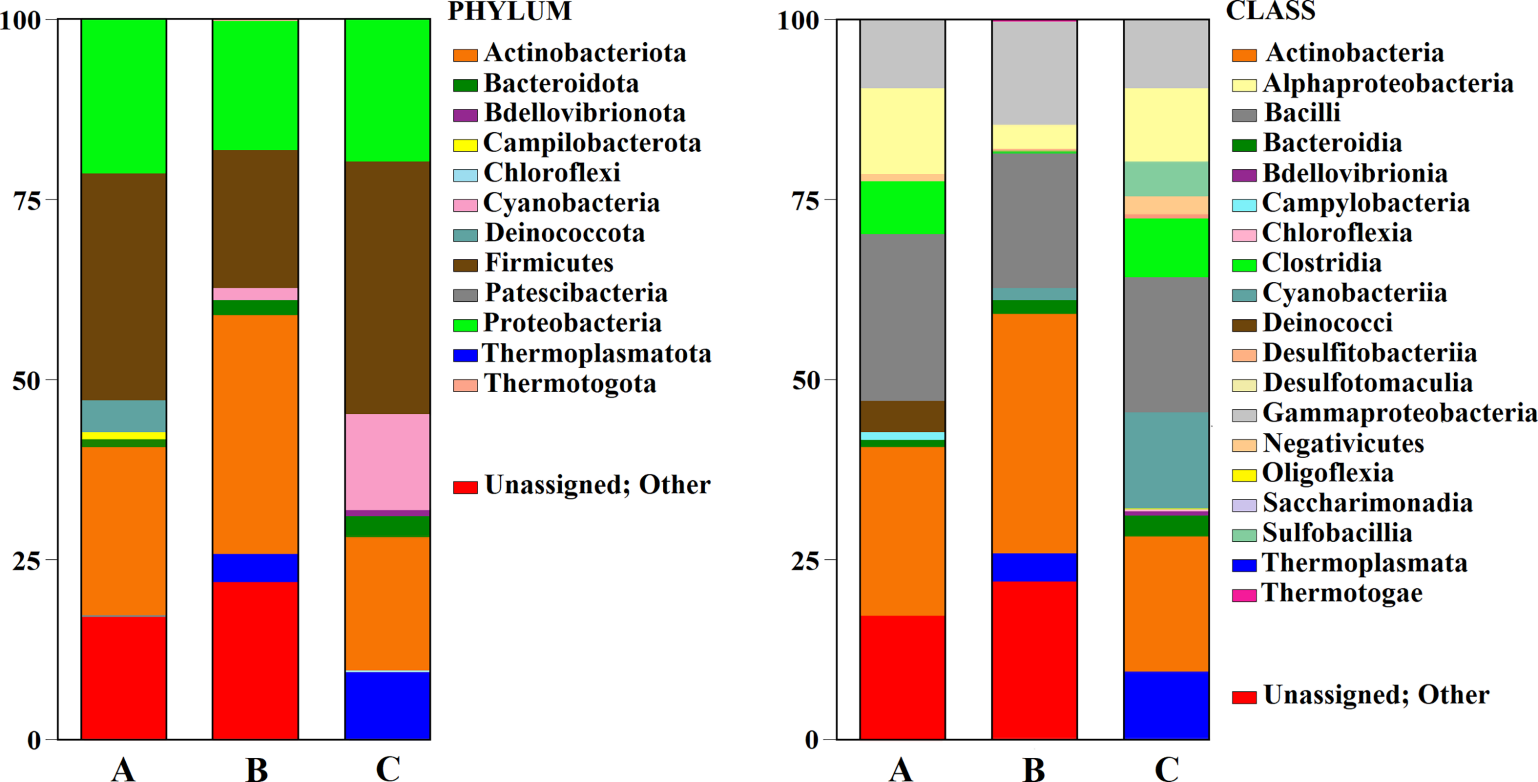
Proportions prokaryotic Operational Taxonomic Units (OTUs) under the phylum and class rank using high‐throughput sequencing data through metabarcoding analysis of 16S rRNA marker in the environmental samples of Mefite for the three sampling sites (A, B, and C; Figure 1). Further details for the taxonomical level are provided in Supplementary Table S3.

**FIGURE 6:**
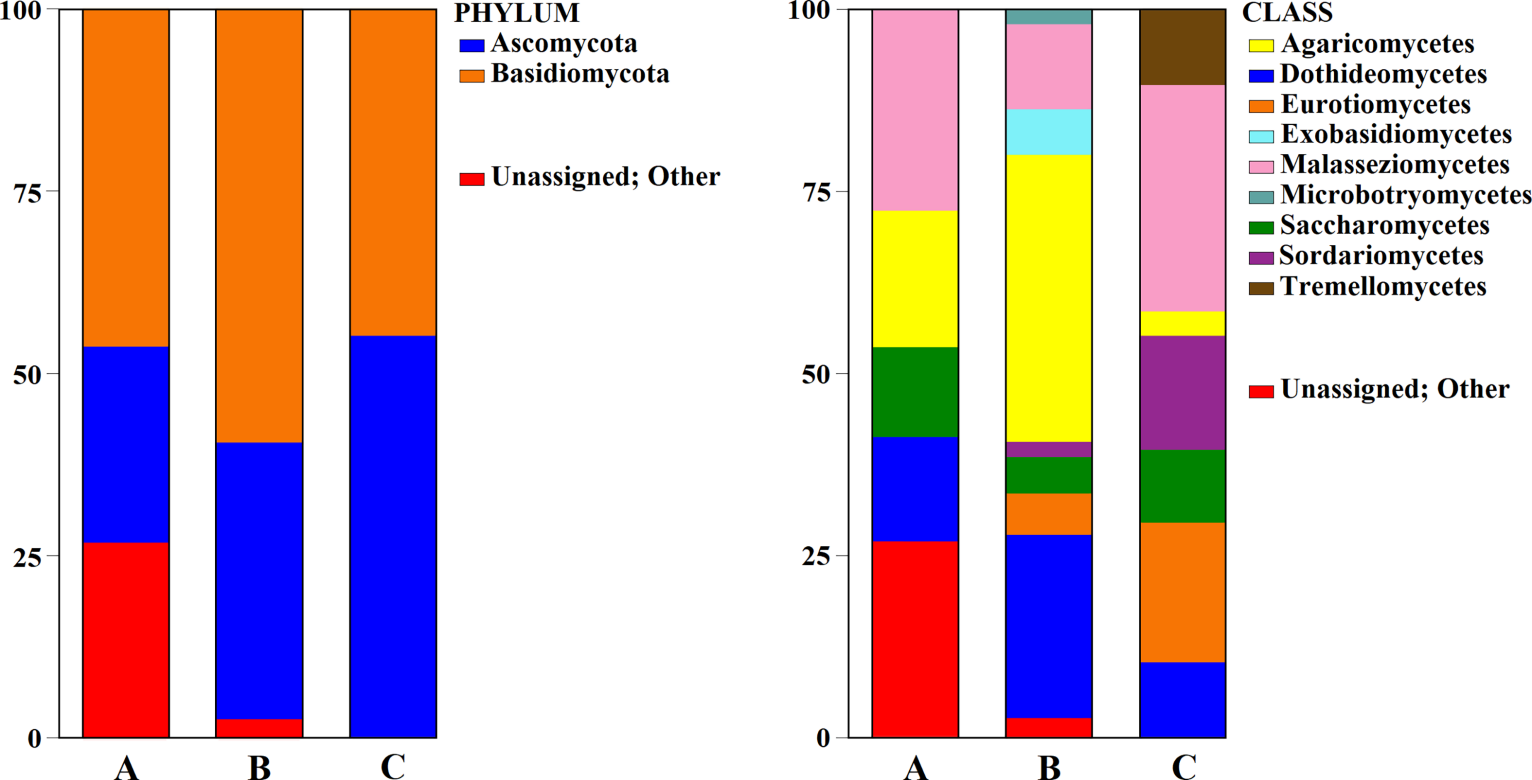
Proportions of fungal Operational Taxonomic Units (OTUs) under the phylum and class rank using high‐throughput sequencing data through metabarcoding analysis of ITS2 nrDNA marker in the environmental samples of Mefite for the three sampling sites (A, B, and C; Figure 1). Further details for the taxonomical level are provided in Supplementary Table S5.

The ANOSIM test, which measures statistical significance among groups by categories (in our case by location), indicated that the difference between locations was close to statistical significance levels (for the 16S rRNA marker, *p*‐value = 0.071; for ITS2, *p*‐value = 0.059). This outcome is likely due to the limited number of replicates. The result is probably attributable to the scarcity of sample replicates (*n* = 2) per location, rather than the dissimilarities between locations. This interpretation is supported by higher values of the ANOSIM test for each marker and by the qualitative composition of OTUs in their bar plots (see Figures 5 and 6).

In the Supplementary Tables ([Link], [Link], [Link], [Link]), the list of all observed OTUs, including both Prokaryotes and Fungi, is provided. Graphical representation of the prokaryotic and fungal OTUs at two taxonomical ranks (phylum and class) for each site is shown in Figures 5 and 6. Results for OTUs exceeding 1% are reported below.

At the phylum prokaryotic level, Firmicutes was the most prevalent taxon in sites A, B, and C, ranging from 35% to 19.2%, with the highest concentrations in C and the lowest in B. This was followed by Actinobacteriota (A: 23.5%, B: 33.2%, C: 18.8%) and by Proteobacteria (A: 21.2%, B: 17.8%, C: 19.7%). The remaining taxa were present in smaller percentages: Bacteroidota (A: 0.9%, B: 1.9%, C: 2.9%); Bdellovibrionota (C: 0.8%); Campilobacterota (A: 1%); Cyanobacteria (B: 1.8%, C: 13.1%); Deinococcota (A: 4.4%); and Thermoplasmatota (B: 3.9%, C: 9.3%). At the class level, the dominant groups were Actinobacteria (A: 23.5%, B: 33.2%, C: 18.8%); Bacilli (A: 23.2%, B: 18.8%, C: 19.2%) and Alphaproteobacteria (A: 11.5%, B: 3.4%, C: 10.1%), followed by Gammaproteobacteria (ranging from 14.4% to 9.6%, higher in B, lower in C); and Bacteroidia (A: 0.9%, B: 1.9%, C: 2.9%); Clostridia (A: 7.2%, C: 7.8%). Some taxa appear in only one or two sites. These included Campylobacteria (A: 1%); Cyanobacteria (B: 1.8%, C: 13.1%); Deinococci (A: 4.4%); Negativicutes (C: 2.7%); Sulfobacillia (B: 4.7%) and Thermoplasmata (B: 3.9%, C: 9.3%). The bacterial microbiota at family level primarily consisted Staphylococcaceae (A: 15.29%, B: 5.89%, C: 10.52%); Streptococcaceae (A: 7.3%, B: 6.9%, C: 3.9%); Corynebacteriaceae (A: 2.6%, B: 12.2%, C: 3.2%); Micrococcaceae (A: 13.7%, B: 4%); Moraxellaceae (A: 5.5%, B: 7.3%, C: 4.7%); Nocardioidaceae (B: 8.1%, C: 0.9%); Rhodobacteraceae (A: 1.3%, C: 4.5%); Sulfobacillaceae (C: 4.8%); Pasteurellaceae (B: 1%, C: 3.5%); Thermaceae (A: 4.4%); Carnobacteriaceae (B: 0.8%, C: 3.6%); Bacillaceae (B: 3.4%); Caulobacteraceae (A: 1.4%, B: 2%); Sphingomonadaceae (C: 2.3%); and Gemellaceae (B: 1.8%, C: 0.8%). Considering the genera found simultaneously in all three analysed sites, even in trace amounts, the bacterial microbiota mainly consists of *Staphylococcus* spp. (A: 15.3%, B: 5.9%, C: 10.5%); *Streptococcus* spp. (A: 7.3%, B: 6.9%, C: 3.9%); *Acinetobacter* spp. (A: 5.5%, B: 7.3%, C: 4.7%); *Corynebacterium* spp. (A: 1%, B: 0.3%, C: 13.4%); *Paracoccus* spp. (A: 1.3%, B: 0.1%, C: 4.5%); *Haemophilus* spp. (A: 0.1%, B: 1%, C: 3.5%). Lastly, the average proportion of unassigned taxa across the sites was 17.3% in A, 21.9% in B, and 0.08% in C.

In the Fungi at the phylum level, Basidiomycota was most present (A 46.3%, B 59.2%, C 44.7%). Ascomycota showed increasing concentration moving from the centre of the pool to the outskirts (A: 26.9%, B: 38%, C: 55.3%). At the class level, within Basidiomycota, Agaricomycetes were observed (A: 18.8%, B: 39.1%, C: 3.3%), Exobasidiomycetes (B: 6.3%), Malasseziomycetes (A: 27.5%, B: 11.7%, C: 30.9%), Microbotryomycetes (B: 2.1%) and Tremellomycetes (C: 10.5%). Among Ascomycota, Dothideomycetes (A: 14.5%, B: 25.3%, C: 10.4%), Eurotiomycetes (B 5.6%, C 19.4%), Saccharomycetes (A 12.4%, B 4.9%, C 9.7%) and Sordariomycetes (B 2.2%, C 15.8%) were noted. At family level, the composition mainly consisted of Malasseziaceae (A: 26.9%, B: 11.7%, C: 26.5%); Debaryomycetaceae (A: 12.4%, B: 4.9%, C 9.7%); Aureobasidiaceae (A: 4.6%, B: 3.2%, C: 10.4%); Chaetomiaceae (C: 15.8%); Aspergillaceae (C: 12.4%); Meruliaceae (B: 9.6%); Pleurotaceae (A: 8.6%); Physalacriaceae (A: 6.4%); Peniophoraceae (B: 6.4%); Corticiaceae (B: 5.8%); Hymenochaetaceae (B: 5.7%); Verrucariaceae (B: 5.6%); Aspergillaceae (C: 4.5%); and Bulleribasidiaceae (C: 4%). The fungal microbiota at the genus level (Table 2), which included taxa concurrently identified in three evaluated sites, comprised *Malassezia* spp. (A: 26.9%, B: 11.7%, C: 26.5%); *Debaryomyces* spp. (A: 12.4%, B: 4.9%, C: 9.7%); *Aureobasidium* spp. (A 4.6%, B 3.2%, C 10.4%). On average, unassigned taxa constituted 27.8% in site A and a smaller proportion of 2.7% in site B.

### Two‐block partial least squares analyses

**FIGURE 7:**
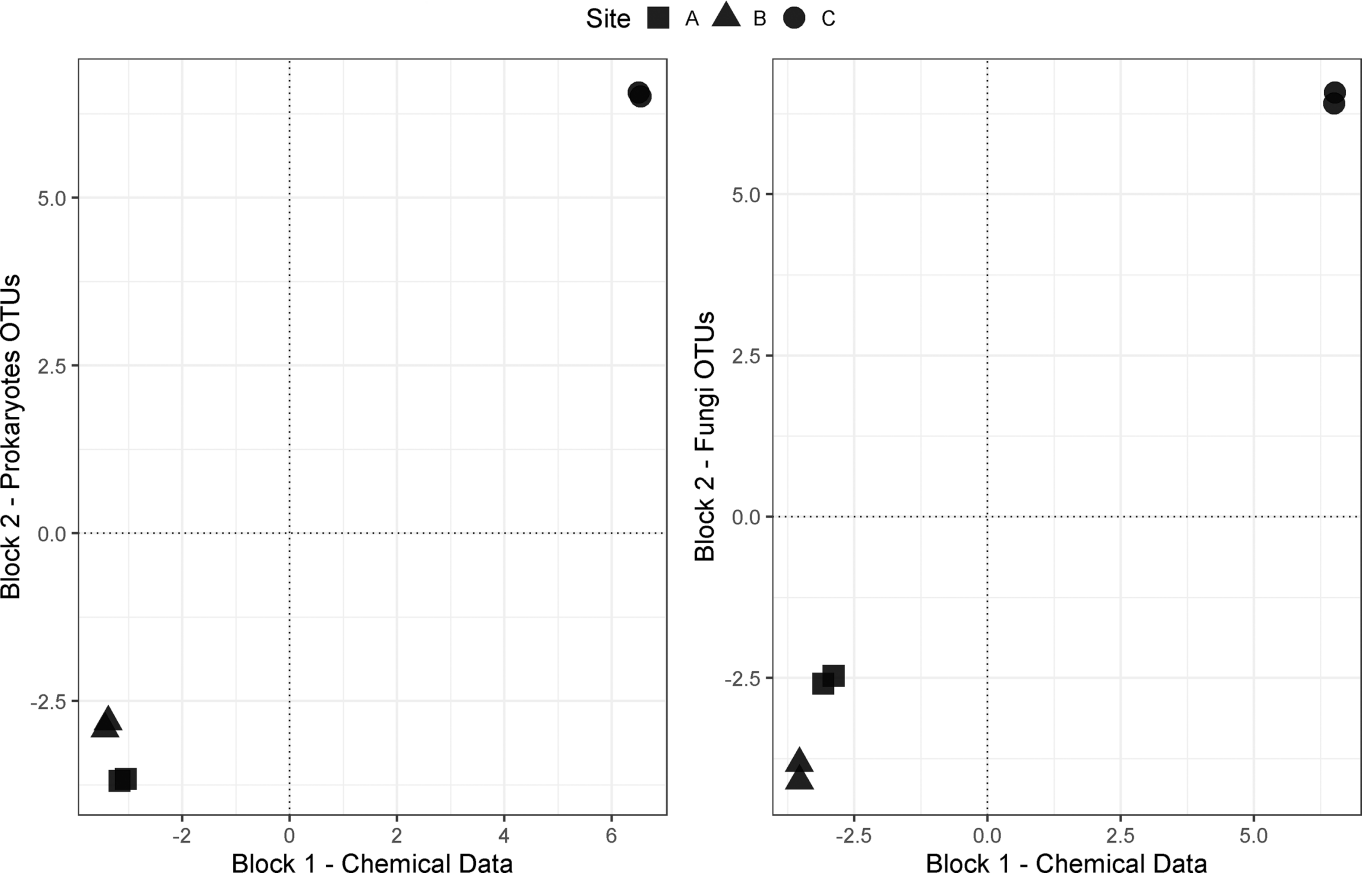
Scatterplots for the first axis of the 2B‐PLS analyses using chemical data versus Prokaryotes OTUs, and chemical data versus Fungi OTUs, respectively. Sampling plots are indicated by different symbols (A with a square, B with a triangle, and C with a circle). Correlations between the different variables within and between blocks are reported in Table 3.

The first axis results of the 2B‐PLS are presented in Figure 7. When both Prokaryotes and Fungi OTUs were analysed against geochemical data, the results clearly showed a distinct separation of site C from sites A and B. Although sites A and B were closer in terms of Prokaryotes OTUs, a gradient from A to B was observed in the analysis of the fungal OTUs. A summary of the correlations within and between blocks for the first axis of the 2B‐PLS can be found in Table 3, with the complete list provided in Supplementary Table S7.

When analysing geochemical data versus Prokaryotes OTUs, site C was differentiated from the others, primarily due to most chemical variables, particularly trace elements like Cr, Al, Li, Pb, V, and others, as well as carbon‐related variables such as cellulose and DOC. F^−^ and NO_2_^−^ were the only anions uniquely associated with site C. These chemical variables were linked to a large number of prokaryotes OTUs. Among those with the highest correlations, OTUs 656,520, 760,967, 1,023,075, 503,315, and 741,701 are notable. These 16S rRNA OTUs are unclassified and warrant further investigation. The remaining OTUs, which showed a high correlation (i.e., correlation ≥0.9) with site C, were predominantly Bacteria, mostly from the Proteobacteria phylum, followed by Firmicutes and Actinobacteriota. In terms of orders, Pseudomonadales was among the most represented.

In sites A and B, which were distinctly from site C, the chemical variables indicated that most anions, particularly SO_4_^2−^ and to a lesser degree, Cl^−^ and NO_3_^−^, were associated with this segregation. This chemical profile correlated with a prokaryotic community characterised by a different group of OTUs. The ones showing the highest correlation (i.e., ≥ 0.9) were OTUs, 1, 3, 12, 181,589, and 5. All of these were Bacteria, except OTU 181589, which was unassigned. Within the Bacteria, they were primarily categorised into the phyla Firmicutes and Actinobacteriota. Firmicutes were divided into the orders Lactobacillales and Veillonellales‐Selenomonadales, whereas Actinobacteriota had OTUs belonging to the order Micrococcales.

In the comparison of geochemical data with fungal OTUs, it was evident that the results for geochemical data closely mirrored those obtained from the analysis of prokaryotic OTUs. Consequently, the trend observed across sites A, B, and C could be described as a variation in fungal OTUs corresponding to the same chemical changes noted for Prokaryotes. Specifically, the Fungi correlated with site C and the concentration of trace elements included one OTU. The OTUs with the highest correlation were 46, 32, 29, 3, and 15,963. All these OTUs belonged to the Ascomycota phylum, with three in the Eurotiales order and two in the Sordariales order. As already mentioned, while locations A and B were clearly separated from location C, when considering fungi OTUs we could see that locations A and B showed a smaller degree of segregation. In detail, OTUs 13, 14, 30, 31, and 33, along with 17 others, were associated with site A. OTU 44 was the primary factor in the segregation of site B. Four OTUs from site B belonged to the Basidiomycota phylum, each within different orders (Cantharellales, Microstromatales, Corticiales, Russulales), and only one to the Ascomycota phylum (order Verrucariales). The key OTU from site A (OTU 44) belonged to the Basidiomycota phylum, within the Polyporales order.

## DISCUSSION

To date, recent studies focusing on organisms that can potentially survive in the cold natural carbon dioxide springs of Mefite are absent. The initial study of the microbial component in Mefite's mud puddle and the surrounding soil was conducted by Totàro‐Aloj (1973), documenting the presence of lithotrophic bacteria, Actynomicetes, and unicellular algae. Subsequent, research on Mefite's algal component identified extremophiles such as *Viridiella fridericiana* P. Albertano, A. Pollio, and R. Taddei (Trebouxiophyceae, Chlorophyta), *Ochromonas vulcania* Gromov, Nikitina and Mamkayeva (Chrysophyceae, Heterokonta), *Cyanidium caldarium* (Tilden) Geitler and *Galdieria sulphuraria* (Galdieri) Merola (Cyanidiophycea, Rhodophyta), as documented in studies by Totàro‐Aloj (1974), Albertano et al. (1991, 1994), and Pinto et al. (1992).

Several studies have been conducted analysing the microbiome of spring water sources around the world. However, research focusing on the composition of thermal mud is less common. Where such studies are available, they focus on the ‘Cyanosphere’ (Gris et al., 2020; Halary et al., 2022). It is important to note that the bacterial and fungal microbiota composition of the Mefite acidic mud samples is strictly related to the chemical and physical characteristics of the site's spring water (Hallberg et al., 2006), also according to recent studies conducted on similar extreme environments (Jaffer et al., 2019; Korzhenkov et al., 2019; Crognale et al., 2022;).

Korzhenkov et al. (2019) reported the results on the water and mud microbiota composition of Parys Mountain (Anglesey, UK), an ecosystem previously exploited for metal mining processes. This area is characterised by low‐to‐moderate temperature (8–18°C) and extreme acidity (pH <2), along with high concentrations of soluble iron and other metals, presenting chemical characteristics closely comparable to those of the Mefite area. Interestingly, in the mud samples, the Thermoplasmata class, along with Archaea, resulted in the highest number of amplicon reads; and recent metagenomic monitoring on extremely acidic sites (pH <3) has confirmed that the largest percentage of the isolated microorganisms are affiliated with mostly uncultured Thermoplasmata taxa (Méndez‐García et al., 2014; Yelton et al., 2013). Korzhenkov et al. (2019) further isolated *Ferrimicrobium acidiphilum* from enrichment cultures, suggesting that the presence of *Ferromicrobium* sp. strains (belonging to the Thermoplasmata class) in our study may be linked to the exploitation of metal ions. Indeed, *F. acidiphilum*, characterised as a mesophilic heterotrophic iron oxidizer and reducer, was also detected at a mine site in North Wales (Johnson et al., 2009), and in sediment columns from Rio Tinto (García‐Moyano et al., 2012). Similarly, in the microbial composition of Mefite muds, Proteobacteria and Actinobacteriota were reported as the most abundant bacterial phyla (Figure 5). This aligns with current knowledge suggesting that these groups include major representatives of acidophilic bacteria (Chen et al., 2016; Mendez‐Garcia et al., 2015). In the CO_2_‐rich (~97% CO_2_) hydrothermal springs of Tenorio Volcano National Park (Costa Rica), Thermoplasmatales and particularly sulphur‐oxidising microorganisms dominate the microbial community. This environment, characterised by high levels of chemical species such as sulphate and iron, is similar to that found in Mefite springs. However, Euryarchaeota, which were the most detected bacterial phylum in the springs of Tenorio Volcano National Park (Arce‐Rodríguez et al., 2019), were not detected in our samples.

A multidisciplinary characterisation of Telese Terme springs and wells at the base of Mount Pugliano Hill (Benevento, southern Italy), a hypothermal area situated 70 km from the Mefite site, revealed a high abundance of the microbial families Sphingomonadaceae and Moraxellaceae (Corniello et al., 2022). Notably, the respective genera *Acinetobacter* and *Sphingomonas* were also detected in the Mefite samples. Crognale et al. (2022) recently published results from research conducted on the hydrothermal, extremely acid Pisciarelli Spring in Campi Flegrei (Naples, southern Italy), located 120 km from the Mefite site. This area, similar to the Mefite sampling location, is characterised by high levels of reduced gaseous species (e.g., H_2_S), very acidic pH values (<3), and near boiling temperatures. Despite the difference in temperatures, several of the most abundant genera identified in Mefite, which are likely involved in Fe‐ and S‐ metabolism, such as *Ferroplasma* spp. and *Sulfobacillus* spp. (Table 1), has been also identified in the extreme environment microbiome of Pisciarelli Spring.

**TABLE 1:** Prokaryotes community at genus levels with a relative total abundance >1%, using high‐throughput sequencing data through metabarcoding analysis of 16S rRNA marker, in the environmental samples of Mefite for the three sampling sites (A, B, C; Figure 1). Further details for taxonomical level are provided in Supplementary Table S4.

Class^Phylum^	Family	Genus	A	B	C
Bacilli^F^	Staphylococcaceae	*Staphylococcus*	15.3	5.89	10.5
Bacilli^F^	Streptococcaceae	*Streptococcus*	7.3	6.9	3.9
Actinobacteria^A^	Corynebacteriaceae	‐	2.6	12.2	3.24
Actinobacteria^A^	Micrococcaceae	*Rothia*	13.7	4	‐
Gammaproteobacteria^P^	Moraxellaceae	*Acinetobacter*	5.5	7.3	4.7
Actinobacteria^A^	Corynebacteriaceae	*Corynebacterium*	1	0.3	13.4
Thermoplasmata^T^	Ferroplasmaceae	*Ferroplasma*	‐	3.9	9.3
Actinobacteria^A^	Nocardioidaceae	*Nocardioides*	‐	8.1	0.9
Alphaproteobacteria^P^	Beijerinckiaceae	*Methylobacterium‐Methylorubrum*	7.1	0.8	‐
Alphaproteobacteria^P^	Rhodobacteraceae	*Paracoccus*	1.3	0.1	4.5
Sulfobacillia^F^	Sulfobacillaceae	*Sulfobacillus*	> 0.01	‐	4.8
Gammaproteobacteria^P^	Pasteurellaceae	*Haemophilus*	0.1	1	3.5
Deinococci	Thermaceae	*Thermus*	4.4	‐	‐
Bacilli^F^	Carnobacteriaceae	*Granulicatella*	‐	0.8	3.6
Negativicutes^F^	Veillonellaceae	*Veillonella*	0.9	‐	2.7
Clostridia^F^	Peptostreptococcales‐Tissierellales	*Anaerococcus*	‐	0.4	3.2
Bacilli^F^	Bacillaceae	*Bacillus*	0.02	3.4	‐
Alphaproteobacteria^P^	Caulobacteraceae	*Brevundimonas*	1.4	2	‐
Actinobacteria^A^	Micrococcaceae	*Micrococcus*	‐	3.1	0.3
Bacteroidia^B^	Prevotellaceae	*Prevotella*	‐	1.9	1.2
Alphaproteobacteria^P^	Sphingomonadaceae	*Sphingomonas*	0.4	‐	2.3
Bacilli^F^	Gemellaceae	*Gemella*	‐	1.8	0.8

*Note*: ^A^Actinobacteriota; ^B^Bacteroidota; ^D^Deinococcota; ^F^Firmicutes; ^P^Proteobacteria; ^T^Thermoplasmatota, ‐no datum.

As for the volcanic origin of the springs and their high carbon dioxide content, Krauze et al. (2017) described a similar environmental condition in the CO_2_‐dominated mofettes of the Cheb Basin (Czech Republic), where CO_2_ values range from 90% to 99%. In these mofettes, the most abundant phyla were Proteobacteria (51.6%) and Actinobacteriota (10.3%), with lesser abundances of Parcubacteria, Omnitrophica, and Chloroflexi. On the other hand, the shallow groundwater profile of the same site showed a predominance of Proteobacteria, Actinobacteriota, Firmicutes, and Bacteroidetes, outlining a profile similar to that of the Mefite mofette (Figure 5), with a comparable abundance in these phyla. Krauze et al. (2017) identified *Gallionella* as the most abundant genus associated with iron‐oxidising processes. In our study, however, there was a prevalence of the iron‐utilising *Ferroplasma* sp. Previous studies have also reported an increase in biomass production of acidophilic, ferritrophic organisms, like *Ferritrophicum*, in response to increased CO_2_ concentrations (Cárdenas et al., 2009; Ni et al., 2018), suggesting that such conditions could be favourable for the growth of iron‐oxidising microorganisms.

Although Li et al. (2011) described the optimal growth temperature for *Sulfobacillus* spp. as being around 50°C, based on isolating the genus from a hydrothermal vent in the Pacific Ocean, it was also detected in our study at a mean value of 1.6% (Table 1). This presence may be linked to the chemical composition of the Mefite site, where sulphate (SO_4_^−2^) is the most concentrated anion. The abundance of this genus in such a sulphide‐rich habitat is associated with its role in sulphur metabolism. Also, in the case of sulphur‐oxidising microorganisms, as for iron‐oxidizers, CO_2−_influenced subsurface systems might play a key role in metals and ions‐rich habitats (Krauze et al., 2017). The presence of the thermophilic genus *Thermus*, comprising 1.5% of the microbiota, was described by Paduano et al. (2018) in their study of thermal springs and muds in Sirmione (Brescia, northern Italy). Similar to *Sulfobacillus* spp., this genus can oxidise sulphur compounds. The relatively high concentrations of *Sulfobacillus* and *Ferroplasma* species in the inner samples may be connected to the enrichment of iron and sulphur species in the area, thus representing the most valuable pathways to gain the most optimised energy yields from the hypothermal sources.

A study by Mosso et al. (1994) evaluating 26 meso‐, hyper‐, and hypo‐thermal mineral springs in Spain revealed the presence of various genera within the bacterial microbiota. These included *Acinetobacter* spp., *Bacillus* spp. (found in 65.4% of the sources), *Staphylococcus* (found in 50% of the sources), and *Micrococcus* spp. Additionally, *Nocardia* spp. and *Corynebacterium* spp. were detected in the microbiota of alkaline hot springs in the Dagestan Republic of Russia, as reported by Khalilova et al. (2014). *Nocardia* spp. was also isolated from mud pool samples in Fiji, a region where the analysis of microbial diversity remains largely unexplored (Pipite et al., 2023). The genus *Thermus* spp. was characterised in the spring water sources of Yellowstone National Park (Munster et al., 1985). Mondal et al. (2022) recently demonstrated the capacity of the bacterial genus *Paracoccus* to withstand very high temperatures, as evidenced by its presence in Himalayan hot springs with temperatures ranging from 78 to 85°C. Interestingly, *Paracoccus* was also identified in the microbiota of the Mefite site (Table 1), despite its lower temperature that classifies the springs as hypothermal, with a range from 10 to 18°C. Additionally, *Sphingomonas* spp., which was found in the Mefite site at a prevalence of 1% (Table 1), was previously described by Lee et al. (2001) in thermal water springs near Taejon (South Korea). *Brevundimonas* spp. and *Rothia* spp., both known for their ability to absorb cadmium, were also isolated from radioactive thermal water samples collected in the Iranian city of Ramsar (Masoudzadeh et al., 2011). It may be therefore hypothesised that the presence of these genera may be related to Cd absorption, leading to concentrations of this element being below detectable limits. In a recent study, Nagarajan et al. (2023), evaluated the microbiota of Taiwan's mud volcanoes, leading to the isolation of *Methylobacter* spp. strains, which were characterised in the present study with a mean prevalence of 2.6% mean value. Furthermore, the bacterial family Carnobacteriaceae, which includes the genus *Granulicatella*, was isolated from warm water samples of the Jaguari River near São Paulo (Brazil) (Becker et al., 2017). Dowidar et al. (1990) also documented the isolation of species from the family Streptococcaceae, notably the genus *Streptococcus*, from well‐water samples in Egypt.

Upon analysing the data at the genus level (Table 1) and comparing the findings available literature, the genera *Staphylococcus* spp. and *Bacillus* spp. were isolated from spring water located in the Maharashtra district (India), as documented by (Jaffer et al., 2019). Furthermore, the presence of these genera in the Mefite samples and in other thermal spring water sources worldwide, typically associated with humans and animals, might be attributed to the presence of animals in these areas. The microbial genera identified in our research (Table 1 and Supplementary Table S3), such as *Acinetobacter*, *Bacillus*, *Micrococcus*, *Nocardia*, *Corynebacterium* and *Streptococcus*, are not typically endemic to geothermal systems, which are usually characterised by an endemic microbiome (Colman et al., 2016; Colman et al., 2021; Fullerton et al., 2021; Power et al., 2018; Rogers et al., 2022). The detection of these genera in geothermal samples could therefore be attributed to either contamination (occurring during sampling or in the laboratory), or to the presence of humans and animals that interact with the springs. In our case, we believe the detection is related to the latter. This is supported by literature noting that the site was/is frequented by animals that often perish there due to the high concentration of CO_2_ (see paragraph ‘Study Site’ for references). Additionally, we employed comprehensive strategies in the laboratory to minimise contaminations, which is corroborated by the absence of these genera in the blank samples we sequenced. This hypothesis gains further from the presence of sequences in the fungal community associated with the genera *Malassezia*, which is commonly linked with animals. Additionally, it is important to note that the only OTUs correlating with the geochemical data characterising the inner samples from the Mefite (samples A and B) are unclassified OTUs (Table 3 and Supplementary Table S7). This observation underscores that the endemic microbiome likely adapted to high‐CO_2_ levels, comprises uncultured groups responsive to geological environmental gradients, as observed and reported by Fullerton et al. (2021). Conversely, the community introduced by external factors, such as human and animal activity or winds and rain, while present and abundant in all samples, does not correlate with changes in geochemical variables.

Analysing mycobiota, differences in fungal composition among the three collected samples were also noted as shown in Figure 6 and Table 2. At the class level, Agaricomycetes were predominantly detected in samples A and B, while Sordariomycetes, Eurotiomycetes, and Tremellomycetes were exclusively found in site C. The presence of these classes and in particular of Sordariomycetes, also identified in the mycobiota of the fracture zones of crystalline bedrock at Olkiluoto in Finland (Sohlberg et al., 2015), could be linked to the ability of some species to thrive in extremely low water levels (Houbraken et al., 2014). At the phylum level, the Mefite hypothermal sites are heavily dominated by members of Ascomycota (approx. 40%) and Basidiomycota (the remaining 60%). This finding aligns with the isolation of these phyla from spring soils reported by Bazzicalupo et al. (2022). Their study, conducted on geothermal soils sampled at Yellowstone National Park (USA), revealed the detection of species from the genus *Talaromyces*, which includes thermophilic and thermotolerant species (Houbraken et al., 2012). *Talaromyces* was identified at the Mefite site with a mean abundance of 0.8% (Supplementary Table S5). Moreover, the Ascomycota phylum has been documented as the dominant fungal group associated with spring sediments globally (Das et al., 2021). The fungal characterisation of the Mefite site led to the detection of common Ascomycota genera, such as *Aspergillus*, *Penicillium*, and *Cladosporium*, which are widespread in spring environments like those in North Sikkim (India) (Das et al., 2021). Additionally, the genus *Flavodon*, detected at a small percentage in Mefite sites A and B (Table 2), had previously been isolated from a coastal marine environment off the west coast of India (Raghukumar et al., 1999). Considering the presence of the *Aureobasidium* genus in the Mefite sites (Table 2), it is noteworthy that Zalar et al. (2008) isolated strains of *Aureobasidium pullulans* (de Bary) G. Arnaud (1918) from seawater samples in the Svalbard Islands (Norway). Species of *Aureobasidium*, mainly found in deep sea environments (Damare et al., 2008), exhibit remarkable resistance to high osmotic pressure, a crucial characteristic for survival in such environments (Sohlberg et al., 2015). In addition, species of the genus *Malassezia*, which are well represented in Mefite samples (Table 2), are often isolated from methane hydrate‐bearing deep‐sea sediments (Lai et al., 2007). These species have been demonstrated to be potentially methylotrophic, and they might play a significant role in converting methane into more accessible carbon and energy substrates. This process can be to be exploited by microbial communities (Lai et al., 2007; Raghukumar et al., 2010; Sohlberg et al., 2015).

**TABLE 2:** Fungi community at genus levels with a relative total abundance >1%, using high‐throughput sequencing data through metabarcoding analysis of ITS2 nrDNA marker, in the environmental samples of Mefite for the three sampling sites (A, B, C; Figure 1). Further details for taxonomical level are provided in Supplementary Table S6.

Class^Phylum^	Family	Genus	A	B	C
Malasseziomycetes^B^	Malasseziaceae	*Malassezia*	26.9	11.7	26.5
Saccharomycetes^A^	Debaryomycetaceae	*Debaryomyces*	12.4	4.9	9.66
Dothideomycetes^A^	Aureobasidiaceae	*Aureobasidium*	4.6	3.2	10.4
Sordariomycetes^A^	Chaetomiaceae	*Chaetomium*	‐	‐	15.8
Eurotiomycetes^A^	Aspergillaceae	*Aspergillus*	‐	‐	12.36
Agaricomycetes^B^	Meruliaceae	*Phlebia*	‐	9.60	‐
Agaricomycetes^B^	Pleurotaceae	*Pleurotus*	8.6	‐	‐
Agaricomycetes^B^	Meruliaceae	*Flavodon*	3.7	3.1	‐
Agaricomycetes^B^	Physalacriaceae	*Cylindrobasidium*	6.4	‐	‐
Agaricomycetes^B^	Peniophoraceae	*Peniophora*	‐	6.4	
Agaricomycetes^B^	Corticiaceae	*Corticium*	‐	5.8	‐
Agaricomycetes^B^	Hymenochaetaceae	*Fuscoporia*	‐	5.7	‐
Eurotiomycetes^A^	Verrucariaceae	*Verrucaria*	‐	5.6	‐
Eurotiomycetes^A^	Aspergillaceae	*Penicillium*	‐	‐	4.5
Tremellomycetes^B^	Bulleribasidiaceae	*Dioszegia*	‐	‐	4

*Note*: ^A^Ascomycota; ^B^Basidiomycota, ‐no datum.

**TABLE 3:** Summary of the correlation between the different variables within and between blocks in the 2B‐PLS analyses (Figure 7). The list of all variables, including the whole list of OTUs, and their correlation is reported in Supplementary Table S7.

Chemical data	Corr.	Prokaryotes OTUs	Chemical data	Corr.	Fungi OTUs
Cr, Al, Li, Pb, V, and other 14 variables	1.0	656,520, 760,967, 1,023,075, 503,315, 741,701, and other 48 OTUs	Pb, Cr, V, Volatile Substances, Al, and other 14 variables	1.0	46, 32, 29, 3, 15,963, and other 8 OTUs
Cellulose, As, and Se	0.9	467,198, 144,048, 516,966, 1,000,547, 356,733, and other 6 OTUs	Cellulose, As, and Se	0.9	
F^−^, DOC, Be, and Mn	0.8	406,064, 521,851, 861,807, and 509,773	F^−^, Be, Mn, and DOC	0.8	7
Te	0.7	341,460	Te	0.7	
NO_2_^−^	0.6		NO_2_^−^	0.6	
	0.5			0.5	
	0.4			0.4	
	0.3	377,874, 321,584, and 30,062		0.3	
	0.2			0.2	9
	0.1	532,569 and 715,102		0.1	
PO4^3−^	0.0	219,151, 495,067 and 617,833	PO4^3−^	0.0	
	−0.1			−0.1	
NO_3_^−^	−0.2	2,751,958	NO_3_^−^	−0.2	
	−0.3	494,906 and 791,149		−0.3	41
	−0.4	252,119, 2,326,547, 539,735, 242,293, 868,615, and other 3 OTUs		−0.4	
Cl^−^	−0.5	967,427, 4,369,229, 1,074,210, 361,100, 268,353, and other 69 OTUs	Cl^−^	−0.5	13, 14, 30, 31, 33, and other 17 OTUs
	−0.6	579,608 and 511,475		−0.6	60
SO_4_^2−^	−0.7	6, 8, and 137,741	SO_4_^2−^	−0.7	
	−0.8	5, 542,475, 496,787, and 1,084,865		−0.8	
	−0.9	1, 3, 12, 181,589, and 5		−0.9	
	−1.0			−1.0	44

The results obtained align with various studies in the literature that focus on the bioenergetic potential of hydrothermal systems, encompassing terrestrial, shallow‐sea, and deep‐sea sites (Lu et al., 2021). It is important to highlight that acidophilic and acid‐tolerant fungi are significant components of extremely acidic habitats (Aguilera & González‐Toril, 2019). Horiike and Yamashita (2015) have shown that acidophilic fungi are capable of resisting heavy metals and can actively sequester high concentrations of these elements. This supports the detection of a wide range of extremophilic fungi in the Mefite sites. Most notably, acidophilic fungi play a crucial role in structured biofilm formation, facilitating metal and mineral precipitation processes, and providing protection for less‐tolerant microorganisms (Aguilera & González‐Toril, 2019).

Finally, an increase in cellulose production was noted from sites A to C, with a more than a twofold increase in concentration. The presence of cellulose in extreme environments has been documented in the past, and several microorganisms inhabiting these habitats are recognised as potential producers of lignocellulolytic and other biotechnological enzymes. These enzymes have enhanced biochemical properties that are essential for industrial bioconversion processes (Thapa et al., 2020). Although fungi are primarily responsible for decomposing the lignocellulosic matrix (Schneider et al., 2012), various bacteria also contribute significantly to this process (López‐Mondéjar et al., 2016). Bacteria such as Firmicutes and Bacteroidetes are known to be key players in degrading biopolymers in different habitats (Dedysh et al., 2006), while bacteria phylogenetically related to *Cytophaga hutchinsonii* Winogradsky (1929) are efficient cellulose degraders in acidic environments such as peat bogs (Pankratov et al., 2011).

In conclusion, the results demonstrate that the physical and chemical characteristics of Mefite mud, including factors like pH, temperature, salts, trace elements, and organic compounds, significantly influence the composition of bacterial and fungal communities. Understanding these communities in previously unexplored sites like the Mefite site is an essential prerequisite for interpreting the biogeochemical interactions that control nutrients and volatile cycling in these systems. This understanding is also crucial for further insight into selective pressures and evolutionary drivers.

## AUTHOR CONTRIBUTIONS

**Olga De Castro:** Conceptualization (lead); data curation (lead); formal analysis (supporting); funding acquisition (equal); investigation (equal); methodology (equal); software (supporting); supervision (lead); validation (equal); visualization (equal); writing – original draft (equal); writing – review and editing (lead). **Mariano Avino:** Formal analysis (lead); methodology (equal); writing – original draft (equal); writing – review and editing (equal). **Federica Carraturo:** Writing – original draft (equal); writing – review and editing (equal). **Emanuela Di Iorio:** Methodology (supporting); writing – original draft (supporting). **Donato Giovannelli:** Formal analysis (supporting); methodology (supporting); writing – original draft (supporting). **Michele Innangi:** Formal analysis (equal); methodology (equal); writing – original draft (equal); writing – review and editing (equal). **Bruno Menale:** Funding acquisition (equal); writing – original draft (equal). **Nicolina Mormile:** Methodology (equal); writing – original draft (equal). **Jacopo Troisi:** Formal analysis (equal); methodology (equal); writing – original draft (equal). **Marco Guida:** Funding acquisition (equal); supervision (equal); validation (supporting); writing – original draft (equal).

## CONFLICT OF INTEREST STATEMENT

The authors declare no conflict of interest.

## Supporting information


**Appendix S1.** Protocol: microbial DNA extraction from mud samples of the Mefite locality.

**Table S1.** Trace element concentration for the three sampling locations (sites A, B, C; Figure 1). All values are μg/g. Values are mean ± standard error of the mean (*N* = 3).
**Table S2.** Anions concentration for the three sampling locations (sites A, B, and C; Figure 1).

**Table S3.** List of prokaryotic Operational Taxonomic Units (OTUs) identified using high‐throughput sequencing data through metabarcoding analysis of 16S rRNA marker, showing relative frequencies per site (duplicates treated as a single sample).

**Table S4.** List of prokaryotic Operational Taxonomic Units (OTUs) identified using high‐throughput sequencing data through metabarcoding analysis of 16S rRNA marker, showing relative frequencies per sample.

**Table S5.** List of fungal Operational Taxonomic Units (OTUs) identified using high‐throughput sequencing data through metabarcoding analysis of ITS2 nrDNA marker, showing relative frequencies per site (duplicates treated as a single sample).

**Table S6.** List of fungal Operational Taxonomic Units (OTUs) identified using high‐throughput sequencing data through metabarcoding analysis of ITS2 nrDNA marker, showing relative frequencies per sample.

**Table S7.** Complete list of the correlation between the different variables within and between blocks in the 2B‐PLS analyses.

## Data Availability

Raw reads are available at NCBI under Bioproject ID PRJNA1031948.
