# Supplementary material for: Profiling microbial communities in an extremely acidic environment influenced by a cold natural carbon dioxide spring: A study of the Mefite in Ansanto Valley, Southern Italy
Source: Environ Microbiol Rep. 2024 Feb 26;16(1):e13241. doi: 10.1111/1758-2229.13241 (PMC10895555; doi:10.1111/1758-2229.13241)
Supplement: Supplementary file 1 — Appendix S1. Protocol: microbial DNA extraction from mud samples of the Mefite locality. [file EMI4-16-e13241-s004.docx]

**Microbial community profiling in an extremely acidic environment interested by cold natural carbon dioxide spring: the case of the Mefite of the Ansanto Valley (southern Italy).**

Olga De Castro, Mariano Avino, Federica Carraturo, Emanuela Di Iorio, Donato Giovannelli, Michele Innangi, Bruno Menale, Nicolina Mormile, Jacopo Troisi, Marco Guida

**Appendix S1**

**Microbial DNA extraction from mud samples of the Mefite locality.**

**(References:** Plassart et al. 2012, De Castro et al. 2015, Lever et al. 2015, update 15.03.2023**)**

1. After to start, weigh **0.24 mg** of **dried** **mud** in a 2 mL tube.

Add **750 µL** of lysis buffer (see appendix) and grind the samples (Ultracool GeneReady; e.g. **Prog. 5** that includes: Shake time 03:00 min; Hold time 01:00 min; T° 6.5°C; Speed 6.50 m/s; Cycle 02) with best spheres for your samples (e.g. spheres of GeneReady Kit Plant PI). Use the "Insert Citation" button to add citations to this document.

**3.** After grinding, add about **1 mL** of lysis buffer.

**4.** Incubate samples at **70°C** for **30 min** in shaker.

**5.** Centrifuge to **7,000xg** for **5 mins** at 20°C.

**6.** After removing the supernatant, proteins were precipitated, with 1/10 volume of 3 M sodium acetate.

**7.** The collected supernatants were incubated for **10 min** at 4°C.

**8.** Centrifuge to **14,000xg** for **5 min**.

**9.** Add **2 volumes** of PE.Na-precipitation buffer (see appendix).

**10.** Let sit in the dark at room temperature for **2 hours**.

**11.** Centrifuge to **14,000xg** for **30 mins** at room temperature.

**12.** Withdraw the supernatant and add **600 μL** of **70% cold Ethanol** and invert once to mix.

**13.** Centrifuge for **10 min** at **14,000xg** at 20°C.

**14.** Repeat step 12, 13.

**15. Dry** the pellet in thermostat to **37 °C** for **10-15 min**.

**16. Resuspend** samples with **400 μL** of **water Molecular Biology Grade (0.2 μm filtered)** and **mix well**.

**17.** Add equal volume **of Chloroform-Isoamyl Alcohol (24:1)** and **mix well** to form an emulsion by shaking tubes with hands.

**18.** Centrifuge for **8 min** at **10,000xg**.

a. *Following centrifugation, you should have three layers: top: aqueous phase, middle: debris and proteins, bottom: Chloroform-Isoamyl Alcohol.*

b. *Proceed to the next step quickly, so the phases do not remix.*

**19.** Pipette off the **aqueous phase (top)** taking care not to suck up any of the middle or chloroform phases.

**20.** Repeat steps 17, 18.

**21.** Place the aqueous phase into a new 1.5 mL tube.

**22.** **Estimate the volume** of the aqueous phase.

**23.** Add **1/10** **volume** of **3 M sodium acetate** and **70%** of **cold isopropanol**. **Mix well**.

**24.** Let sit in freezer (-20°C) for **1 hour**.

**25.** Centrifuge for **15 min** at maximum speed.

Orient tubes in an equal fashion to facilitate subsequent removal of supernatant without disturbing resultant DNA pellet.

**26.** **Pipette** off the liquid, being **careful** not to lose the pellet with your DNA. The DNA pellet at this

stage is very loose and difficult to see.

**27.** Add **500 μL** of **70% cold Ethanol** and invert once to mix.

**28.** Centrifuge for **10 min** at maximum speed.

**29.** **Pipette** off the liquid, being **careful** not to lose the pellet with your DNA.

**30.** Repeat steps 28, 29.

**31.** **Dry** the pellet in thermostat to **37 °C** for **10-15 min**.

**32.** **Resuspend** samples with **10 μL** of **water Molecular Biology Grade filtered (0.2 μm; e.g., Ambion AM9937)**. For a good resuspension it is advisable to vortex well.

**Appendix [all solution must be autoclaved (120°C- 20’) or filtered (0.2 μm) according to the system study]:**

**Lysis Buffer ISOm**: for 250 mL (Plassart et al., 2012);

- 25 mL of 1 M Tris, pH 8 (= 100 mM);
- 50 mL of 0.5 mM EDTA, pH 8 (= 100 mM);
- 5 mL of 5 M NaCl or 1.461 g (= 100 mM);
- to 180 mL with H_2_0 Molecular Biology Grade (optional: 0.2 μm filtered) and autoclave;
- after, 5 g SDS (= 2% w/v);
- 5 g of PVP 40 kDa (= 2% w/v);
- To 250 mL with water filtered and/or autoclaved.

**PE.Na-precipitation buffer ^†^:** for 10 mL (Lever et al., 2015);

- 3 g of PEG-8000 (= 30% w/v);
- 3 mL 5M NaCl or 0,87 g (= 1.5 M);
- to 10 mL with H_2_0 Molecular Biology Grade sterile or filtered (0.2 μm).

**^†^**, In Lever et al. (2015) is used 1.6 M NaCl. We used 1.5 M NaCl because it is also present in the lysis buffer.

**References**

De Castro, O., Gianguzzi, L., Carucci, F., De Luca, A., Gesuele, R. & Guida M. (2015) Old sleeping Sicilian beauty: seed germination in the palaeoendemic *Petagnaea gussonei* (Spreng.) Rauschert (Saniculoideae, Apiaceae). *Plant Biology*, 17, 1095–1098. doi: 10.1111/plb.12333.

Lever, M.A., Torti, A., Eickenbusch, P., Michaud, A.B., Šantl-Temkiv, T. & Jørgensen B.B. (2015) A modular method for the extraction of DNA and RNA, and the separation of DNA pools from diverse environmental sample types. *Frontiers in Microbiology*, 6, 476. doi: fmicb.2015.00476. eCollection 2015.

Plassart, P., Terrat, S., Thomson, B., Griffiths, R., Dequiedt, S., Lelievre, M., Regnier, T., Nowak, V., Bailey, M., Lemanceau, P., Bispo, A., Chabbi, A., Maron, P.A., Mougel, C. & Ranjard L. (2012) Evaluation of the ISO Standard 11063 DNA extraction procedure for assessing soil microbial abundance and community structure. *PlosOne* **7**: 44279. doi: 10.1371/journal.pone.0044279.
