# Supplementary material for: Profiling microbial communities in an extremely acidic environment influenced by a cold natural carbon dioxide spring: A study of the Mefite in Ansanto Valley, Southern Italy
Source: Environ Microbiol Rep. 2024 Feb 26;16(1):e13241. doi: 10.1111/1758-2229.13241 (PMC10895555; doi:10.1111/1758-2229.13241)
Supplement: Supplementary file 2 — Table S1. Trace element concentration for the three sampling locations (sites A, B, C; Figure 1). All values are μg/g. Values are mean ± standard error of the mean (N = 3). Table S2. Anions concentration for the three sampling locations (sites A, B, and C; Figure 1). [file EMI4-16-e13241-s002.docx]

**Microbial community profiling in an extremely acidic environment interested by cold natural carbon dioxide spring: the case of the Mefite of the Ansanto Valley (southern Italy).**

Olga De Castro, Mariano Avino, Federica Carraturo, Emanuela Di Iorio, Donato Giovannelli, Michele Innangi, Bruno Menale, Nicolina Mormile, Jacopo Troisi, Marco Guida.

**Supplementary TABLES S1-S2**

| **Table S1.** Trace element concentration for the three sampling locations (sites A, B, and C; Figure 1). All values are µg/g. Values are mean ± standard error of the mean (N=3). | | | | |
| --- | --- | --- | --- | --- |
|  | **A** | **B** | **C** |  |
| **Al** | 5083.217±100.368 | 4601.217±159.245 | 17355.81±191.426 |  |
| **As** | 0.423±0.057 | 0.43±0.068 | 0.993±0.062 |  |
| **B** | 4.563±0.058 | 4.817±0.101 | 11.167±0.06 |  |
| **Ba** | 26.367±0.589 | 18.077±2.958 | 50.637±3.117 |  |
| **Be** | 0.06±0.006 | 0±0.008 | 0.107±0 |  |
| **Co** | 0.653±0.029 | 0.63±0.024 | 1.4±0.031 |  |
| **Cr** | 3.987±0.084 | 3.693±0.113 | 13.06±0.14 |  |
| **Cu** | 22.457±0.324 | 21.45±1.275 | 28.593±1.243 |  |
| **Fe** | 1749.263±26.444 | 1510.02±70.143 | 4255.987±90.85 |  |
| **Hg** | 1.093±0.043 | 0.963±0.049 | 2.183±0.044 |  |
| **Li** | 1.203±0.007 | 1.2±0.012 | 3.403±0.009 |  |
| **Mn** | 15.87±0.117 | 15±0.582 | 17.113±0.576 |  |
| **Ni** | 41.143±0.079 | 36.94±1.833 | 85.883±1.548 |  |
| **Pb** | 1.247±0.035 | 1.113±0.058 | 2.76±0.062 |  |
| **Se** | 0.11±0.025 | 0.097±0.027 | 0.16±0.036 |  |
| **Sn** | 0.157±0.009 | 0.133±0.01 | 0.427±0.017 |  |
| **Sr** | 27.1±0.63 | 20.047±2.039 | 79.473±2.313 |  |
| **Te** | 0.16±0 | 0.2±0.017 | 0.193±0.015 |  |
| **Tl** | 0.023±0.003 | 0.02±0.003 | 0.063±0.003 |  |
| **U** | 0.087±0.007 | 0.07±0.009 | 0.233±0.003 |  |
| **V** | 4.993±0.064 | 4.867±0.035 | 16.333±0.012 |  |
| **Zn** | 3.273±0.07 | 3.117±0.032 | 6.033±0.064 |  |

| **Table S2.** Anions concentration for the three sampling locations (sites A, B, and C; Figure 1). All values are mg/L. Values are mean ± standard error of the mean (N=3). | | | |
| --- | --- | --- | --- |
|  | **A** | **B** | **C** |
| **Cl^-^** | 88.45±1.022 | 87.567±1.022 | 88.153±1.022 |
| **F^-^** | 3.333±0.033 | 3.700±0.133 | 3.967±0.12 |
| **NO_2_^-^** | 0.21±0.001 | 0.213±0.001 | 0.213±0.001 |
| **NO_3_^-^** | 13.617±0.133 | 13.307±0.223 | 13.44±0.205 |
| **PO_4_^3-^** | 121.353±0.407 | 122.977±1.076 | 123.79±1.073 |
| **SO_4_^2-^** | 733.303±4.953 | 750.64±7.43 | 735.78±6.553 |
